# Isolated splenic metastasis of endometrial cancer 12 years after treatment: A case report and literature review

**DOI:** 10.1097/MD.0000000000029178

**Published:** 2022-05-06

**Authors:** Xiali Teng, Min Jiang, Xiaowei Zhu, Rongrong Dou, Donglan Yuan, Junxing Huang, Hong Yu

**Affiliations:** a Department of Pathology, Taizhou People's Hospital Affiliated to Dalian Medical University, Taizhou, Jiangsu, China; b Department of Gynecology, Taizhou People's Hospital Affiliated to Dalian Medical University, Taizhou, Jiangsu, China; c Department of Oncology, Taizhou People's Hospital Affiliated to Dalian Medical University, Taizhou, Jiangsu, China.

**Keywords:** endometrial cancer, splenectomy, splenic metastasis

## Abstract

**Rationale:**

The spleen is an uncommon metastatic organ for malignant solid tumors because of its special anatomy and microenvironment. Isolated splenic metastasis of endometrial cancer is an extremely rare clinical event, with only 17 cases reported in literature.

**Patient concerns:**

We report the case of a 58-year-old woman with abdominal distension and nausea for 7 months who had undergone surgery and chemotherapy for endometrioid adenocarcinoma 12 years previously. A space-occupying lesion in the upper pole of the spleen was observed on an abdominal ultrasound.

**Diagnosis:**

The spleen was resected, and splenic metastasis of endometrial adenocarcinoma was histologically confirmed.

**Interventions:**

Splenectomy was performed, and no lymph nodes or other metastases were observed. The patient received postoperative chemotherapy with 6 cycles of docetaxel and carboplatin.

**Outcomes:**

The patient recovered well 11 months postoperatively, with no evidence of recurrence or metastatic disease.

**Lesson:**

Since the time interval between the diagnosis of primary endometrial cancer and splenic metastasis may be very long, it may be necessary to monitor the recurrence of endometrial cancer after primary treatment.

## 1 Introduction

Endometrial cancer is a common malignant tumor of the female reproductive system.^[1]^ For endometrial cancer, the main route of metastasis is lymphatic spread, and distant metastasis beyond the pelvic area may also occur. The common distant metastatic sites for endometrial cancer are the lungs, liver, and bones.^[2]^ Metastases to the spleen from solid cancers are uncommon, with a prevalence of 2.3% to 7.1%.^[3]^ Although splenic metastasis arises from gastric cancer, colorectal cancer, lung cancer, nasopharyngeal carcinoma, ovarian cancer, breast cancer, and other types of cancer,^[4–8]^ isolated splenic metastasis from endometrial adenocarcinoma is extremely rare. To date, only 17 such cases have been reported in the literature. Here, we report a case of endometrial cancer with isolated metastasis to the spleen 12 years after hysterectomy.

## 2 Case presentation

A 58-year-old woman underwent total abdominal hysterectomy and bilateral salpingo-oophorectomy 12 years previously, with a histopathological diagnosis of moderately differentiated endometrioid adenocarcinoma involving the muscle layer. After surgery, the patient was treated with adjuvant sequential chemotherapy consisting of 6 cycles of paclitaxel and carboplatin. The patient exhibited good tolerance to chemotherapy and no obvious adverse effects were observed. The patient remained asymptomatic until July 2020.

**Figure 1:**
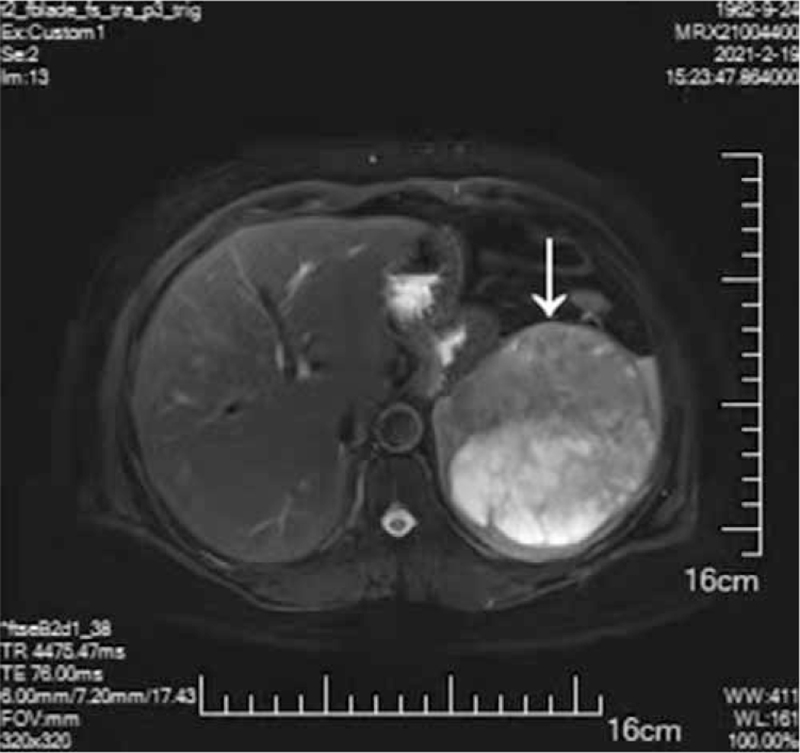
MRI image of the liver and spleen shows a space-occupying lesion of the spleen. MRI = magnetic resonance imaging.

In July 2020, she presented with abdominal distension and nausea. Abdominal ultrasonography revealed a space-occupying lesion in the upper pole of the spleen. Subsequent magnetic resonance imaging confirmed the presence of a mass measuring 11.7 × 10.0 × 8.6 cm (Fig. 1) in February 2021. Laboratory data revealed elevated serum levels of carbohydrate antigen (CA) 19-9 (636.45 U/mL) and CA125 (180.90 U/mL) with no other changes. After careful pre-operative assessment, the patient was scheduled for the operative removal of the splenic mass in February 2021. During surgery, the lesion was found to be isolated splenic cancer. Splenectomy was performed because a careful examination of the abdomen showed no lymph nodes or other metastases.

**Figure 2:**
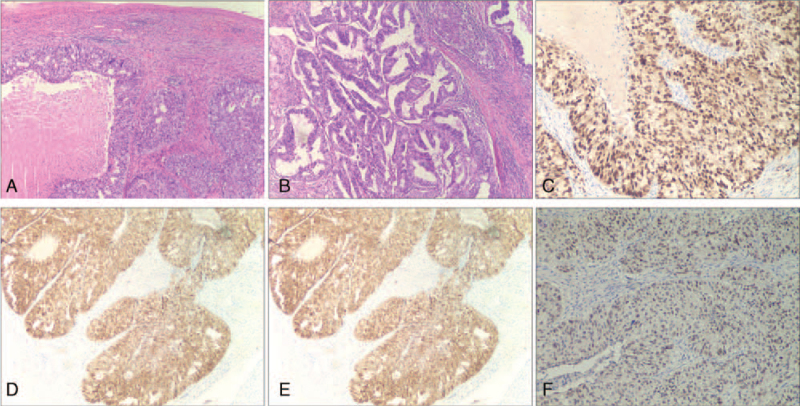
Histopathological analysis. A. The interface between normal splenic tissue and metastatic adenocarcinoma (40×). B. The tumor cells show a glandular tube-like structure and grow invasively (100×). C. Diffuse and strong ER expression in cancer cells (100×). D. Diffuse and strong PR expression in cancer cells (100×). E. Diffuse and strong vimentin expression in cancer cells (100×). F. Diffuse and strong PAX-8 expression in cancer cells (100×).

Gross pathology showed that the mass (10.0 × 9.5 × 7.0 cm) with irregular borders almost completely occupied the splenic parenchyma. Paraffin-embedded sections confirmed the diagnosis of high-grade endometrial adenocarcinoma (Fig. 2). Immunohistochemical analysis revealed that the cancer cells were positive for CK20, vimentin, epithelial membrane antigen, PAX8, P16, P53, estrogen, and progesterone receptors, and negative for CDX2, SATB-2, WT-1, IMP3, and CK7. The Ki-67 index was approximately 60% (Fig. 2F). The tumor is proficient in mismatch repair.

After successful removal of the tumor, a dramatic decline in serum CA19-9 level was observed (124.08 U/mL). The patient subsequently received adjuvant sequential chemotherapy with 6 cycles of docetaxel and carboplatin. The patient had no obvious adverse effects and recovered well within an 11-month follow-up period.

## 3 Discussion

The spleen is an uncommon metastatic organ for malignant solid tumors because of its special anatomy and microenvironment. Isolated splenic metastasis of endometrial cancer is an extremely rare clinical event, with only 17 cases reported in literature.^[9–25]^ It was first reported by Kopacz et al^[9]^ in 1970 (because this article is a Polish paper, we were unable to obtain its abstract or full text). The details of the 18 documented cases (including this case) of splenic metastasis from endometrial cancer are shown in Table 1.

**Table 1:** Clinical features of 18 cases of splenic metastasis from endometrial cancer.

Reference	Age (yrs)	Clinical features	Endometrial cancer: stage and treatment	Interval from hysterectomy and tumor size	Treatment	Metastasis of other sites	Follow-up
^[9]^							
^[12]^	66	Asymptomatic	IAG2, TH + BSO, pelvic RT + BT	20 mos, 8-cm splenic mass	Splenectomy, RT to splenic bed	No	Abdominal metastases at 31 mos DOD
^[13]^	59	Left hypochondrial pain, splenomegaly	IAG3, TH + BSO	11 mos (spleen measured 20 × 4 × 8 cm and weighted 2050 g)	Splenectomy, hormone therapy	No	Colon metastases at 10 mos DOD
^[14]^	72	Left hypochondrial pain, splenomegaly	IBG2, TH + BSO	33 mos, 10-cm splenic mass	Splenectomy	No	Abdominal metastases at 6 mos DOD
^[15]^	52	Vaginal bleeding, left hypochondrial pain	IIIA, TH + BSO	12 mos, 6-cm splenic mass	Pre-operative chemotherapy, splenectomy, postoperative chemotherapy	Pelvic recurrence, invasion of the bladder	Local bladder recurrence and reoperation at 9 mos NED
^[16]^	62	Left hypochondrial pain, splenomegaly	IB, TH + BSO, pelvic RT	12 mos, 10-cm splenic mass	Splenectomy, hormone therapy, RT to splenic bed	No	Abdominal metastasis at 6 mos NED
^[17]^	47	Asymptomatic	III, TH + BSO, chemotherapy, pelvic RT	73 mos, routine US and CT detected a splenic mass	Splenectomy	No	28 mos NED
^[18]^	55	Vaginal bleeding	IG2, TH + BSO + BPLND	28 mos, 6-cm splenic mass	Pelvic RT and BT, splenectomy, chemotherapy	Vaginal vault	12 mos NED
^[19]^	62	Left hypochondrial pain, splenomegaly	IIG1, TH + BSO + BPLND	72 mos, spleen measured max 21 cm	Splenectomy	No	Not recorded
^[20]^	49	Left hypochondrial pain, splenomegaly	IBG2, TH + BSO, pelvic RT	43 mos, 7.5-cm splenic mass	Splenectomy, chemotherapy	No	46 mos NED
^[21]^	43	Splenomegaly	TH + BSO, hormone therapy	120 mos, 5-cm splenic mass	Splenectomy, hormone therapy	No	Follow-up time not recorded NED
^[22]^	60	Asymptomatic	II, TH + BSO, pelvic RT	18 mos, 6-cm spleen mass	Splenectomy	No	18 mos NED
^[23]^	58	Asymptomatic	IIBG2, TH + BSO + BPLND, pelvic RT + BT	18 mos, 10-cm spleen mass	Pre-operative chemotherapy, splenectomy, postoperative chemotherapy	Lung	6 mos NED
^[24]^	54	Asymptomatic	IAG3, TH + BSO, pelvic RT + chemotherapy	18 mos, 6.5-cm spleen mass	Splenectomy, chemotherapy	No	The pelvic cavity recurred at 2 mos and disappeared after pelvic radiotherapy and chemotherapy NED
^[25]^	54	Left hypochondrial pain	IAG2, TH + BSO	50 mos, 9-cm spleen mass	Splenectomy, chemotherapy	No	Follow-up time not recorded NED
^[26]^	69	Left hypochondrial pain	IAG3, TH + BSO + BPLND	32 mos, 9-cm spleen mass	Splenectomy	No	Follow-up time not recorded NED
^[10]^	57	Left hypochondrial pain, splenomegaly	IIIC, TH + BSO	20 mos, 12-cm spleen mass	Splenectomy, chemotherapy	No	Follow-up time not recorded NED
Our case	58	Asymptomatic	IAG2, TH + BSO, chemotherapy	12 yrs, 10-cm spleen mass	Splenectomy, chemotherapy	No	5 mos NED

BPLND = bilateral pelvic lymph node dissection, BSO = bilateral salpingo-oophorectomy, BT = brachytherapy, DOD = died of disease, NED = no evidence of disease, RT = radiotherapy, TH = total hysterectomy.

The average and median ages at diagnosis of splenic metastasis were 57.4 and 57 years, respectively. Only 1 case reported by Andrei et al^[10]^ was high-grade serous adenocarcinoma, whereas the others were endometrioid adenocarcinoma. All patients underwent at least a total hysterectomy and bilateral salpingo-oophorectomy. There were 10 cases of stage I, 3 cases of stage II, 3 cases of stage III, and 1 case of an unknown stage. Nine patients received adjuvant therapy after hysterectomy, including external pelvic radiotherapy (3 cases), external pelvic radiotherapy and brachytherapy (2 cases), external pelvic radiotherapy and chemotherapy (2 cases), hormone therapy (1 case), and chemotherapy alone (our case). The interval between the initial diagnosis of endometrial cancer and splenic metastasis ranged from 11 to 120 months, and the mean interval was 36.25 months in a previous study. In this case, the longest interval from endometrial cancer diagnosis to splenic metastases was approximately 12 years. In terms of clinical symptoms and signs, 6 patients (35.3%) presented with left hypochondrial pain and clinically obvious splenomegaly; 2 patients (11.8%) presented with left hypochondrial pain but had no clinically significant obvious splenomegaly; 1 patient (5.9%) had no obvious discomfort, but clinical examination revealed obvious splenomegaly; and 2 patients (11.8%) presented with vaginal bleeding due to recurrence at the vaginal wall but had no obvious splenomegaly. The remaining 6 cases (35.3%) were asymptomatic and had no clinically obvious splenomegaly, and the splenic mass was detected occasionally by imaging studies during routine follow-up. Fourteen patients (82.4%) developed solitary splenic metastasis. The remaining 3 cases (17.6%) were concomitant with other metastatic sites at the same time, of whom 2 had pelvic metastases and 1 had lung metastases. The splenic metastatic mass measured 5 to 12 cm, with an average size of 8 cm. Metastatic tumors were located in the splenic parenchyma without involvement of the splenic capsule. All patients underwent splenectomy, and 12 patients received adjuvant treatment after splenectomy, including 8 cases of chemotherapy, 2 cass of oral progesterone treatment, 1 case of splenic bed radiotherapy, and 1 case of splenic bed radiotherapy and oral progesterone treatment. The follow-up period after splenectomy ranged from 2 to 46 months; at the end of follow-up, 10 patients (58.8%) were alive without disease, 3 patients (17.6%) were alive with recurrence or metastasis at other sites, 3 patients (17.6%) died of abdominal metastasis, and 1 patient (5.9%) did not record the outcome.

Splenic metastasis from cancers of the female reproductive system typically originates from ovarian cancer.^[11]^ Ovarian cancer metastasizes to the spleen mostly through peritoneal spread; thus, splenic metastasis from ovarian cancer is more often part of a disseminated disease. In contrast to ovarian cancer, splenic metastasis of endometrial cancer is extremely rare. Splenic metastasis from endometrial cancer is usually solitary and limited to the splenic parenchyma. This indicates that endometrial cancer metastasizing to the spleen occurs predominantly via the hematogenous route. Approximately 35% of patients with splenic metastasis from endometrial cancer do not present with typical positive symptoms and signs, such as left hypochondrial pain and/or obvious splenomegaly. These patients were incidentally detected during routine follow-up imaging studies. Therefore, it is easy to miss patients with splenic metastasis from endometrial cancer. Our patient had symptoms such as nausea and vomiting during the course of the disease, which was easily misdiagnosed as gastrointestinal disease. Splenic metastasis from endometrial cancer is detected early, mostly depending on medical history and imaging studies, and the final diagnosis depends on histomorphology and immunohistochemistry. The interval between the diagnosis of endometrial cancer and splenic metastasis was prolonged, ranging from 11 months to 12 years. This indicates that regular clinical examinations and follow-up imaging studies are required after the initial treatment of endometrial cancer. In all cases, the patients underwent splenectomy, which not only relieved the pain caused by splenomegaly but also prevented serious complications such as splenic rupture and splenic vein thrombosis.

In conclusion, solitary splenic metastasis from endometrial cancer is extremely rare and the metastatic mass is usually limited to the splenic parenchyma. Since patients with splenic metastasis may be asymptomatic and the time interval between the diagnosis of primary endometrial cancer and splenic metastasis may be very long, this disease is easily missed and misdiagnosed by clinicians. Therefore, careful and extended follow-up after primary treatment is critical in patients with endometrial cancer. When a splenic occupying lesion is found but the pre-operative diagnosis is unknown, the disease should be considered. Once splenic metastasis is diagnosed, splenectomy should be performed as soon as possible, and adjuvant therapy, including chemotherapy, radiotherapy, and targeted therapy, can be added to extend the survival time of patients within the bounds.

## Author contributions

**Conceptualization:** Xiali Teng, Hong Yu.

**Data curation:** Rongrong Dou, Donglan Yuan.

**Formal analysis:** Xiali Teng, Min Jiang, Hong Yu.

**Investigation:** Xiali Teng, Min Jiang, Hong Yu.

**Methodology:** Xiaowei Zhu.

**Resources:** Hong Yu.

**Writing – original draft:** Xiali Teng, Min Jiang.

**Writing – review & editing:** Junxing Huang, Hong Yu.
